# Capacitance-Based Frequency Adjustment of Micro Piezoelectric Vibration Generator

**DOI:** 10.1155/2014/484381

**Published:** 2014-07-15

**Authors:** Xinhua Mao, Qing He, Hong Li, Dongliang Chu

**Affiliations:** ^1^School of Energy Power and Mechanical Engineering, North China Electric Power University, Beijing 102206, China; ^2^Henan Institute of Science and Technology, Xinxiang 453003, China

## Abstract

Micro piezoelectric vibration generator has a wide application in the field of microelectronics. Its natural frequency is unchanged after being manufactured. However, resonance cannot occur when the natural frequencies of a piezoelectric generator and the source of vibration frequency are not consistent. Output voltage of the piezoelectric generator will sharply decline. It cannot normally supply power for electronic devices. In order to make the natural frequency of the generator approach the frequency of vibration source, the capacitance FM technology is adopted in this paper. Different capacitance FM schemes are designed by different locations of the adjustment layer. The corresponding capacitance FM models have been established. Characteristic and effect of the capacitance FM have been simulated by the FM model. Experimental results show that the natural frequency of the generator could vary from 46.5 Hz to 42.4 Hz when the bypass capacitance value increases from 0 nF to 30 nF. The natural frequency of a piezoelectric vibration generator could be continuously adjusted by this method.

## 1. Introduction

With the development of monitoring technology, wireless sensor networks are widely applied in medical monitoring, structural health monitoring, and industrial equipment condition monitoring and other fields. At present, it has many problems in supplying power for wireless sensor network nodes. For example, batteries have limited lifetime and serious pollutions. Power lines have high costs. In order to fundamentally solve the power supply problem of wireless sensor network nodes, many scholars have been trying to extract vibration energy from the environment and convert it into electricity. Because a cantilever piezoelectric vibration generator has a simple structure, small volume, low cost, and high output voltage, it becomes a research hotspot for converting vibration energy into electric energy [1–12].

A cantilever piezoelectric vibration generator is sensitive to frequency changes of environmental vibration sources. If environmental frequency deviates 2~3 Hz from the natural frequency of piezoelectric generator, it will lead to output power sharply dropping [13–19]. In order to improve its adaptation environmental ability, a cantilever piezoelectric vibration generator is designed usually to add external mechanical device for changing the structure or stiffness of the beam. The purpose is to adjust its natural frequency. However, it would increase the piezoelectric generator volume and production difficulty and also raise the cost of production by this method. The semiactive synchronous switch circuit was adopted to adjust the generator stiffness [20]. It could achieve to regulate the natural frequency. But the control circuit itself would consume energy, which would reduce the output power of the vibration generator. A multicantilever and single-mass piezoelectric generator was designed [21]. The piezoelectric generator has a lot of natural frequencies but brings out new manufacturing difficulties. It would cause high production costs.

For solving the above problems, on the basis of the relationship of the piezoelectric capacitance Young's modulis, capacitance FM technology is adopted to turn the bending stiffness of the piezoelectric beam in this paper. The purpose is to adjust the natural frequency. Different frequency regulation schemes are designed. FM effects of different schemes are simulated. The authors have analyzed structure parameters of piezoelectric generator influence on the frequency adjusting range. Experiments have been carried out to verify FM effects.

## 2. Capacitance FM Principle and Theory Model of a Micro Piezoelectric Generator

### 2.1. Capacitance FM Principle

The relationship of natural frequency *ω*, stiffness *K*, and mass *M* is described as follows:

(1) $$\begin{array}{c} \omega =\sqrt{\frac{K}{M}}. \end{array}$$

**Figure 1:**
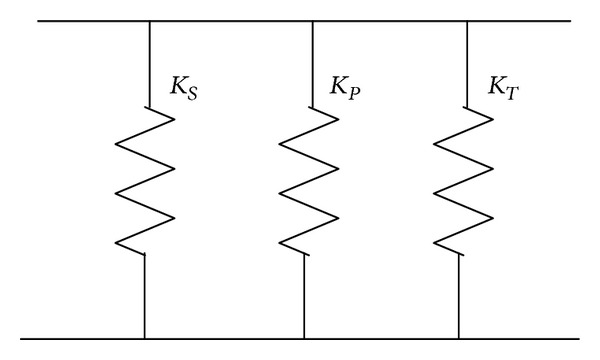
The equivalent stiffness of piezoelectric beam.

The mass of a piezoelectric generator is unchanged after being manufactured. If you want to change its natural frequency, you need to change the stiffness of a piezoelectric generator. According to the literature [22–24], we found the following relations:

(2) $$\begin{array}{c} K=K_{S}+K_{P}+K_{T}=K_{S}+K_{P}+{\theta^{\prime }}^{T}{C^{\prime }}^{-1}\theta^{\prime }, \end{array}$$

where *K*_*S*_ is the stiffness of the elastic substrate, *K*_*P*_ is the mechanical stiffness of the piezoelectric layer, *K*_*T*_ is the electromechanical coupling stiffness, *C*′ is the piezoelectric capacitance, and *θ*′ is electromechanical coupling coefficient. The equivalent connection type is shown in Figure 1.

According to (2), we found that the system stiffness can change when electromechanical coupling or capacitance value of the piezoelectric layer is adjusted. Because of its characteristics being decided by the piezoelectric layer material characteristic and geometrical feature of the piezoelectric beam, the electromechanical coupling term is not easy to change. Therefore, the natural frequency is adjusted only by changing the piezoelectric capacitance values.

**Figure 2:**
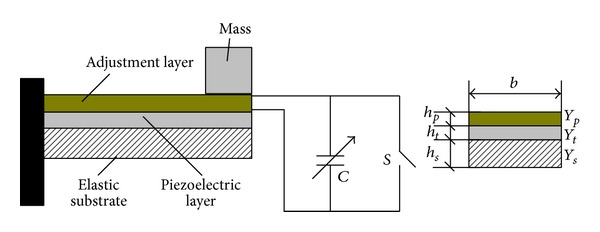
Capacitance FM principle.

**Figure 3:**
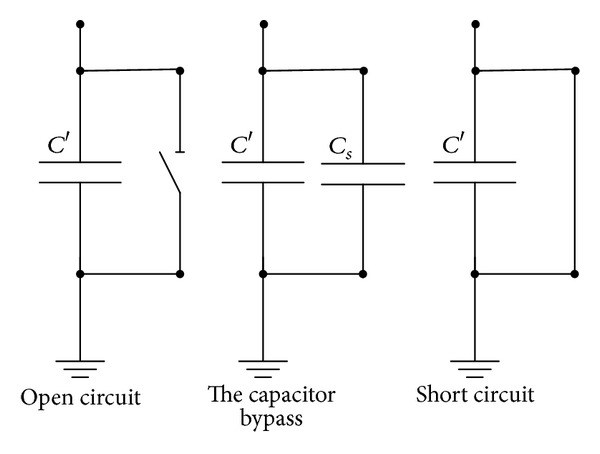
Frequency regulation method with shunt capacity.

We can design an adjustment layer for adjusting the stiffness by the above situation. A bypass capacitor is lain aside the adjustment layer; it is showed in Figure 2. The natural frequency of the system can be adjusted with the bypass capacitance changes. Frequency regulation method with shunt capacity is showed in Figure 3.

The total capacitance of the system equals the sum of the bypass capacitance and the piezoelectric capacitance. When *C*_*S*_ = *∞*, *K*_*T*_ = 0. Equivalent stiffness of the piezoelectric beam is the smallest, and the natural frequency is the lowest. When *C*_*S*_ = 0, *K*_*T*_ = *∞*. The natural frequency would be maximized. Equivalent stiffness of the piezoelectric beam is the biggest, and the natural frequency is the highest. From the above analysis, we know that the adjusting range of the natural frequency is decided by short circuit and open circuit states of the bypass capacitor.

### 2.2. Capacitance FM Scheme and Theory Model

**Figure 4:**
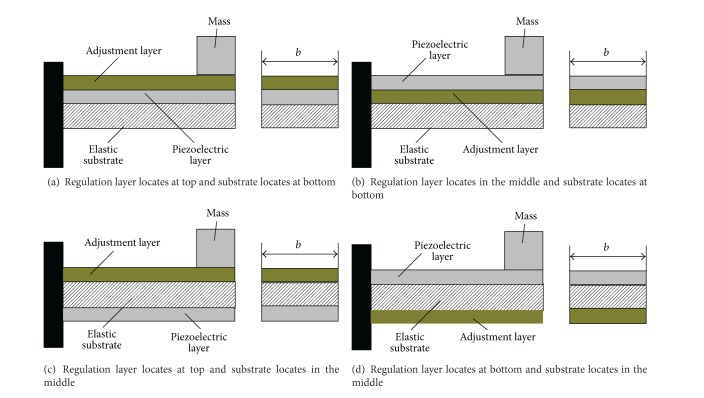
Schemes of the piezoelectric generator based on capacitor FM.

By various position relations among the adjusting layer, the piezoelectric layer, and the substrate, FM schemes of a piezoelectric generator have the following four cases based on capacitance FM technique. It is shown in Figure 4.

The bending stiffness of a piezoelectric generator is [25–27]

(3) $$\begin{array}{c} YI=Y_{s}I_{s}+Y_{p}I_{p}+Y_{t}I_{t}, \end{array}$$

where *Y*_*s*_ *I*_*s*_ is the bending stiffness of the substrate, *Y*_*p*_ *I*_*p*_ is the bending stiffness of the piezoelectric layers, and *Y*_*t*_ *I*_*t*_ is the bending stiffness of the piezoelectric adjusting layer.

The young modulus of the adjustment layer is as follows:

(4) $$\begin{array}{c} Y_{t}={\left(s_{11}^{E}-\frac{d_{31}^{2}bl}{h_{t}C^{z}}\right)}^{-1}, \end{array}$$

where *C*_*z*_ equals to the sum of the piezoelectric capacitor *C*_*t*_ and the parallel capacitor *C*_*S*_.

According to (2) and (3), (1) can be written as

(5) $$\begin{array}{c} \omega =\lambda_{r}^{2}\sqrt{\frac{Y_{s}I_{s}+Y_{p}I_{p}+I_{t}{\left(s_{11}^{E}-d_{31}^{2}bl/h_{t}\left(C_{t}+C_{s}\right)\right)}^{-1}}{M}}. \end{array}$$

In scheme A, elasticity modulus of the piezoelectric beam in neutral axis position is described as follows [28–32]:

(6) Y−=npbhpy−p+ntbhty−t+bhsy−snpbhp+ntbht+bhs=nphp(hs+ht+hp/2)+ntht(hs+ht/2)+hs2/2nphp+nht+hs,

where *h*_*t*_, *h*_*p*_, and *h*_*s*_, respectively, are the thicknesses of the piezoelectric layer, the adjustment layer, and elastic substrate; *n*_*t*_, *n*_*p*_, and *n*_*s*_, respectively, are the elastic modulus ratios of the piezoelectric layer, the adjustment layer, and elastic substrate; $\overset{-}{Y}$ is the neutral axis position of the piezoelectric beam; ${\overset{-}{y}}_{t}$, ${\overset{-}{y}}_{p}$, and ${\overset{-}{y}}_{s}$, respectively, are the neutral axis positions of the piezoelectric layer, the adjustment layer, and elastic substrate;inertia moment is described as follows:

(7) Ip=Ip′+Apdp2=112npbhp3+npbhp(hs+ht+hp2−Y−)2,It=It′+Atdt2=112ntbht3+ntbht(hs+ht2−Y−)2,Is=Is′+Asds2=112bhs3+bhs(hs2−Y−)2,

where *A*_*t*_, *A*_*s*_, respectively, are cross-sectional areas of the piezoelectric layer and elastic substrate; *d*_*t*_, *d*_*p*_, *d*_*s*_, respectively, are the distances among the neutral axis.

In scheme B, the elasticity modulus and inertia moment of the piezoelectric beam are described as follows:

(8) Y−=ntbhty−t+npbhpy−p+bhsy−sntbht+npbhp+bhs=ntht(hs+hp+ht/2)+nphp(hs+hp/2)+hs2/2nht+nphp+hs,It=It′+Apdp2=112ntbht3+ntbht(hs+hp+ht2−Y−)2Ip=Ip′+Apdp2=112npbhp3+npbhp(hs+hp2−Y−)2Is=Is′+Asds2=112bhs3+bhs(hs2−Y−)2.

This has the same elasticity modulus and inertia moment of the piezoelectric beam in scheme C and scheme D; it is described as follows:

(9) Y−=npbhpy−p+bhsy−s+ntbhty−tnpbhp+bhs+ntbht=nphp(ht+hs+hp/2)+hs(ht+hs/2)+nt(ht2/2)nphp+hs+nht,

(10) Ip=Ip′+Apdp2=112npbhp3+npbhp(ht+hs+hp2−Y−)2Is=Is′+Asds2=112bhs3+bhs(ht+hs2−Y−)2It=It′+Apdp2=112ntbht3+ntbht(ht2−Y−)2.

The above-mentioned (5)~(10) aretheory models of different schemes based on capacitance FM technology.

## 3. Simulation and Analysis Based on FM Technology

### 3.1. The Adjusting Range Analysis of Different Schemes

**Figure 5:**
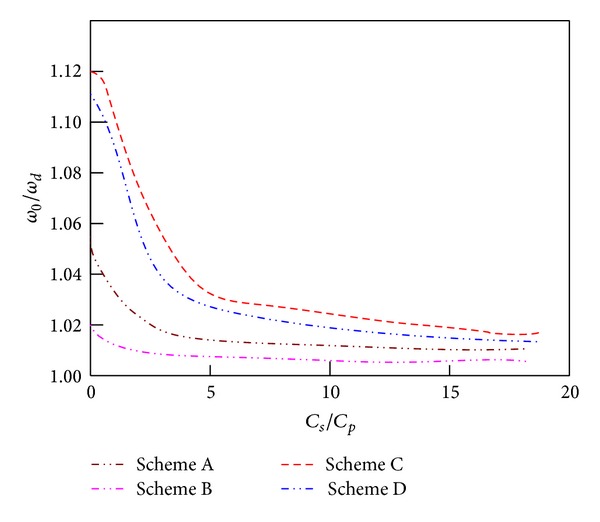
Natural frequency ratio versus capacity ratio.

According to the above equations from (5) to (10), we could obtain rules of the natural frequency with the bypass capacitance changes. It is shown in Figure 5. The figure shows that the natural frequency of the piezoelectric beam would gradually reduce with the increase of the bypass capacitor value. When the bypass capacitor is continuously adjusted, we could obtain arbitrary frequency between the open-circuit frequency and the closed-circuit frequency. Frequency adjusting ranges are close and the largest in schemes C and D. When 0 < *C*_*s*_/*C*_*p*_ < 5, the natural frequency has obvious adjustment effects by adjusting bypass capacitors. And when *C*_*s*_/*C*_*p*_ < 5, it hardly has any effects. According to the above analysis, if making frequency adjustment range the largest, there is not only a suitable capacitor FM scheme but also a reasonable ratio of the bypass capacitance to the piezoelectric capacitance.

### 3.2. Structure Parameters of a Piezoelectric Beam Effect on the FM Range

**Figure 6:**
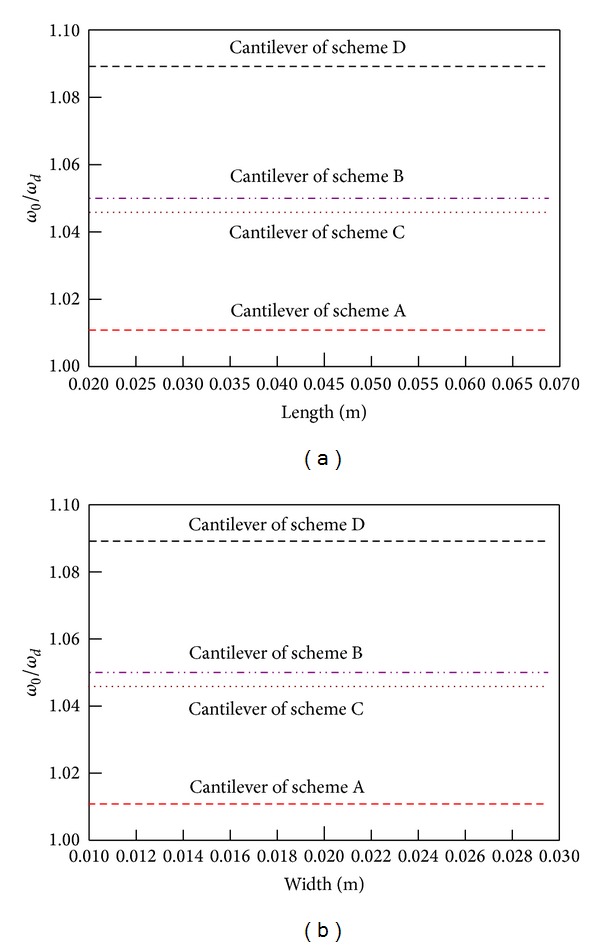
Capacity frequency regulation range versus length and width.

In order to study the effect of piezoelectric generator geometry on the capacitor FM technology, material properties of the cantilever remain the same. Keep the cantilever thickness unchanged and analyze the influence of length change and width change on the frequency tuning range. It is shown in Figure 6. The figures show that changes of the length and width of the piezoelectric beam have no any effects on the natural frequency adjusting range.

**Figure 7:**
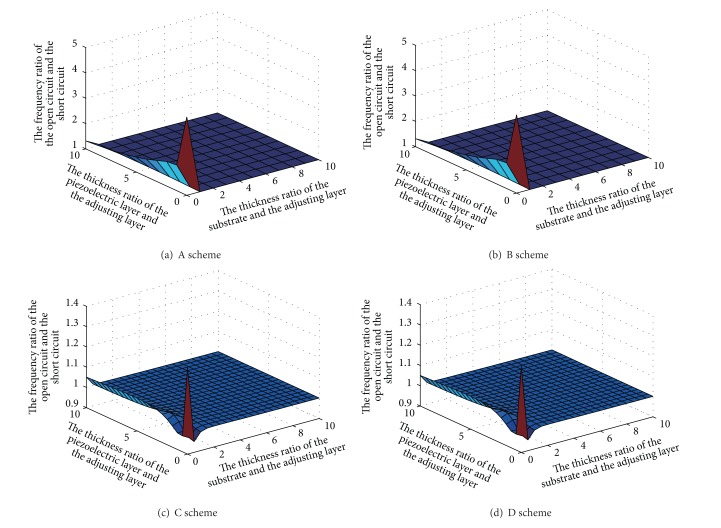
Natural frequency ratio versus thickness ratio.

Keep the piezoelectric adjustment layer thickness unchanged, and adjust the thicknesses of the substrate and the piezoelectric layer. We could obtain the rules of the natural frequency with the piezoelectric beam thickness ratio changes. It is shown in Figure 7.

The figures show that the smaller thickness ratio, the larger the natural frequency adjustable range. This is because the fact that the piezoelectric adjustment layer is relatively thicker when the thickness ratio is smaller, and stiffness adjustable range is relatively larger. Stiffness adjustment characteristics are affected by vibration. Stiffness ratio would fall sharply. When anyone of two thickness ratios is greater than 5, adjustable ranges of the natural frequency would fast become small in schemes A and B. When anyone of two thickness ratios is greater than 2, adjustable ranges of the natural frequency would fast become small in schemes C and D.

## 4. Experiments and Result Analysis

**Figure 8:**
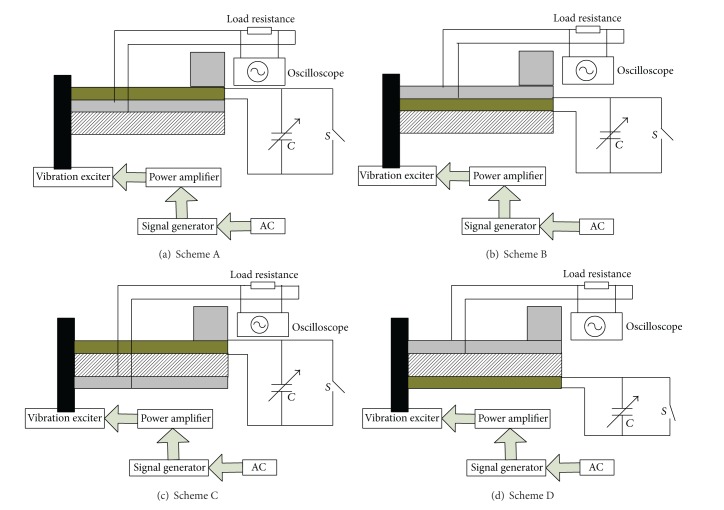
Experiment scheme based on capacitance FM.

**Figure 9:**
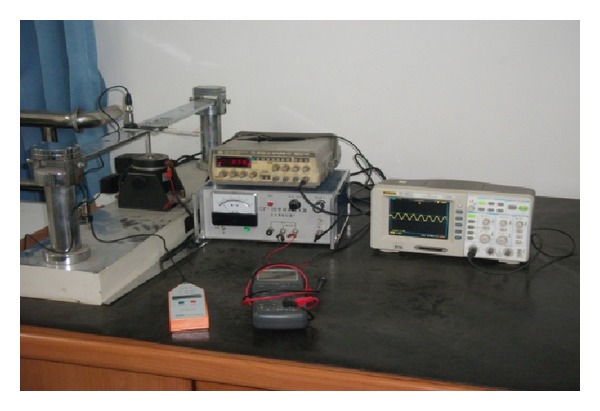
Experimental platform of piezoelectric generator.

**Figure 10:**
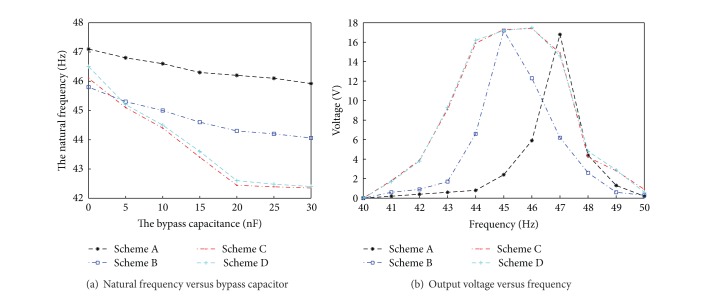
Experimental results of capacitance FM technology.

Experimental schemes are shown in Figure 8. The cantilever of micro piezoelectric vibrating generators used in the experiment is 65 mm long, 5 mm wide, and 0.5 mm thick. The mass size is 5 mm long, 5 mm wide, and 5 mm thick. And its density is 11340 kg/m^3^. The load resistance is 100 Ω. The acceleration of the vibration exciter is 2 m/s^2^. An experimental platform of the piezoelectric generator is shown in Figure 9. Adjustment ranges of the bypass capacitor are from 0 to 30 nF. When the bypass capacitor is opened or closed, adjust the signal frequency and observe the oscilloscope. Initial natural frequencies of different schemes would be obtained. When the switch *S* is disconnected, adjusting the bypass capacitor, the bypass capacitance value would increase gradually from small to big. Capacitance FM ranges and out voltages of different generators are shown in Figure 10.

The experimental results show that the natural frequency is 45.9 Hz in short circuit and 47.1 Hz in open circuit in scheme A. The adjustable frequency range is about 1.2 Hz. The natural frequency is 44.1 Hz in short circuit and 45.8 Hz in open circuit in scheme B. The adjustable frequency range is about 1.7 Hz. The natural frequency is 42.3 Hz in short circuit and 46.2 Hz in open circuit in scheme C. The adjustable frequency range is about 3.9 Hz. The natural frequency is 42.4 Hz in short circuit and 46.5 Hz in open circuit in scheme D. The adjustable frequency range is about 4.1 Hz. So, schemes D and C are close and better than schemes A and B. These results are consistent with the previous theoretical analysis. When bypass capacitance is less than 20 nF, the natural frequency could continuously change by adjusting the bypass capacitance. When the bypass capacitance is more than 20 nF, the natural frequency of the piezoelectric generator is essentially the same. This may be due to the fact that the ratio of the bypass capacitance to the piezoelectric capacitance exceeds 5. It is an unreasonable ratio.

## 5. Conclusions

In order to make the natural frequency of a piezoelectric generator approaching the environment frequency, the capacitance FM technology is adopted to adjust the natural frequency of piezoelectric generator in this paper. Different FM schemes are designed, and the FM theory model has been built. This has carried out the theoretical analysis and numerical simulation. The main research conclusions are as follows.

1. The natural frequency of piezoelectric generator could be continuously adjusted by capacitance FM technology. However, the adjusting range is not larger. It needs to be used in concert with other adjustment frequency methods.
2. In order to get maximum FM range, it needs not only suitable capacitance FM scheme but also the reasonable ratio of bypass capacitance to piezoelectric capacitance.
3. Adjusting range of the natural frequency has nothing to do with piezoelectric beam length and width. It is closely related to thicknesses among the adjustment layer, the piezoelectric layer, and the substrate.
